# Pharmacoinformatics-Based Approach for Uncovering the Quorum-Quenching Activity of Phytocompounds against the Oral Pathogen, *Streptococcus mutans*

**DOI:** 10.3390/molecules28145514

**Published:** 2023-07-19

**Authors:** Shakti Chandra Vadhana Marimuthu, Jayaprabhakaran Murugesan, Ewa Babkiewicz, Piotr Maszczyk, Murugesan Sankaranarayanan, Esakkimuthu Thangamariappan, Joseph Christina Rosy, Sureshbabu Ram Kumar Pandian, Selvaraj Kunjiappan, Vanavil Balakrishnan, Krishnan Sundar

**Affiliations:** 1 Department of Biotechnology, Kalasalingam Academy of Research and Education, Krishnankoil 626126, India; chandravadhana16@gmail.com (S.C.V.M.); prabhakaran.jp.15398@gmail.com (J.M.); temlight8@gmail.com (E.T.); christinarosy.j@gmail.com (J.C.R.); srkpandian@klu.ac.in (S.R.K.P.); selvaraj.k@klu.ac.in (S.K.); b.vanavil@klu.ac.in (V.B.); 2 Department of Hydrobiology, Faculty of Biology, University of Warsaw, 02-089 Warsaw, Polandp.maszczyk@uw.edu.pl (P.M.); 3 Biological and Chemical Research Centre, University of Warsaw, 02-089 Warsaw, Poland; 4 Medicinal Chemistry Research Laboratory, Department of Pharmacy, Birla Institute of Technology and Science-Pilani, Pilani 333031, India; murugesan@pilani.bits-pilani.ac.in

**Keywords:** *Streptococcus mutans*, dental caries, homology modeling, molecular docking, molecular dynamics simulation

## Abstract

*Streptococcus mutans*, a gram-positive oral pathogen, is the primary causative agent of dental caries. Biofilm formation, a critical characteristic of *S. mutans*, is regulated by quorum sensing (QS). This study aimed to utilize pharmacoinformatics techniques to screen and identify effective phytochemicals that can target specific proteins involved in the quorum sensing pathway of *S. mutans*. A computational approach involving homology modeling, model validation, molecular docking, and molecular dynamics (MD) simulation was employed. The 3D structures of the quorum sensing target proteins, namely SecA, SMU1784c, OppC, YidC2, CiaR, SpaR, and LepC, were modeled using SWISS-MODEL and validated using a Ramachandran plot. Metabolites from *Azadirachta indica* (Neem), *Morinda citrifolia* (Noni), and *Salvadora persica* (Miswak) were docked against these proteins using AutoDockTools. MD simulations were conducted to assess stable interactions between the highest-scoring ligands and the target proteins. Additionally, the ADMET properties of the ligands were evaluated using SwissADME and pkCSM tools. The results demonstrated that campesterol, meliantrol, stigmasterol, isofucosterol, and ursolic acid exhibited the strongest binding affinity for CiaR, LepC, OppC, SpaR, and Yidc2, respectively. Furthermore, citrostadienol showed the highest binding affinity for both SMU1784c and SecA. Notably, specific amino acid residues, including ASP86, ARG182, ILE179, GLU143, ASP237, PRO101, and VAL84 from CiaR, LepC, OppC, SecA, SMU1784c, SpaR, and YidC2, respectively, exhibited significant interactions with their respective ligands. While the docking study indicated favorable binding energies, the MD simulations and ADMET studies underscored the substantial binding affinity and stability of the ligands with the target proteins. However, further in vitro studies are necessary to validate the efficacy of these top hits against *S. mutans*.

## 1. Introduction

*Streptococcus mutans* is a gram-positive bacterium commonly found in the human oral cavity. While it is considered part of the normal microbial flora in the oral cavity, it is also the primary causative agent of dental caries [1,2]. Despite numerous studies that have been reported and implemented in the past to mitigate dental caries, none of them have succeeded in completely eradicating the disease. Additionally, *S. mutans* is known to be associated with extra-oral pathological conditions such as infective endocarditis, IgA nephropathy, cerebral microbleeds, and atherosclerosis [3,4]. Therefore, there is a pressing need to develop a practical approach for effectively eliminating *S. mutans*. The Darwinian theory postulates that organisms develop resistance to factors that threaten their survival. This phenomenon is a primary driver behind the development of antibiotic resistance in several pathogens [5]. Hence, it is imperative to develop a treatment that can selectively inhibit the pathogen’s virulence without endangering its overall survival.

One such strategy is controlling the quorum sensing of bacteria. Quorum sensing is a density-dependent bacterial communication mechanism that regulates biofilm formation and the expression of virulent characteristics. Therefore, targeting this mechanism may control their virulence without affecting their survival. Several virulence factors of *S. mutans* are involved in quorum sensing mechanisms, and targeting these factors may impair cell communication [6,7].

In the sequenced oral pathogen, *S. mutans* UA159, the CiaRH two-component signal transduction system (TCS) acts as a global regulator for multiple stress responses, including biofilm formation, acid tolerance, bacteriocin production, genetic competence, stress resistance, and pathogenesis [8]. Bacterial oligopeptide transport systems (Opp) play a role in regulating ATP hydrolysis and energy generation required for solute transport [9]. The Sec translocase and yidC are translocase systems that assist in the transport of proteins across the cell membrane [10]. Another virulent protein of *S. mutans* is encoded by the gene smu1784c, which is involved in stress management and biofilm formation [11].

Regularly used chemical substances such as fluoride, quaternary ammonium salts, and other commonly used antimicrobial agents can lead to undesirable side effects [12,13]. Prolonged use of antimicrobial medications has also contributed to the emergence of antimicrobial resistance, which is now recognized as a significant health problem [14,15]. Consequently, there has been a growing interest in exploring the potential of natural herbal plants as quorum-quenching agents to combat pathogens [16,17,18]. These natural products offer many advantages, such as reduced costs, fewer side effects, and increased efficacy [19]. For example, green tea extract has been found to suppress the growth of *Porphyromonas gingivalis*, and its potential in preventing and treating periodontitis is being investigated [15]. Furthermore, aqueous extracts of *Azadirachta indica* (Neem) sticks have been shown to inhibit bacterial aggregation, proliferation, adherence to hydroxyapatite, and synthesis of insoluble glucan, thus altering in vitro plaque development [20]. To expedite the process of identifying molecules with desired antibacterial activity from plant extracts, a time-saving approach is needed. Therefore, computational methods are utilized to accelerate the drug development process. Pharmacoinformatics tools play a crucial role in the rapid discovery of drugs for various diseases, offering a cost-effective approach to drug discovery. Several computer-based tools can be employed to search for potential drug targets in pathogens such as *S. mutans* and others.

Furthermore, these tools also aid in the study of drug–target interactions. In the current study, drug targets involved in the quorum sensing of *S. mutans* have been selected based on our previous study [21]. An in silico approach has been undertaken to screen potential phytochemicals from selected plants and to analyze their binding potential with the target proteins.

## 2. Results

### 2.1. Homology Modeling and Model Validation of the Target Proteins

SWISS-MODEL performs BLAST for each target protein against PDB and provides a list of templates. In the present study, for all the seven target proteins, model structures were built using SWISS-MODEL since none of them had a crystal structure available. For each of the selected target proteins, five templates were selected based on the identity and sequence coverage, and the 3D models were built.

The modeled structures that were built were assessed and validated using a Ramachandran plot using the SWISS-MODEL structure assessment tool, and one out of five models was selected for further studies (Supplementary Figure S1). The predicted structures of all the target proteins are depicted in Supplementary Figure S2.

### 2.2. Molecular Docking

Among the 110 ligands tested, 17 compounds were found to bind effectively with all the target proteins (Table 1). Among the 17 compounds that exhibited higher binding towards the selected targets of *S. mutans*, 15 compounds were from *A. indica*, and 1 each from *M. citrifolia* and *S. persica*. Among the 17 lead compounds, the top hits that exhibited lower binding energy and, hence, high binding affinity against each target were then selected for further simulation analysis (Table 2).

**Figure 1:**
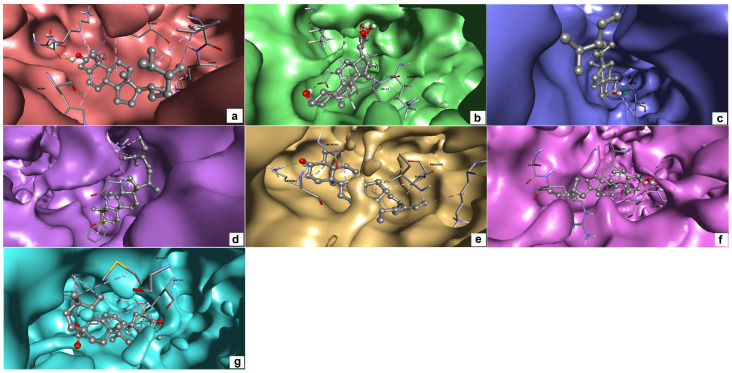
3D interaction of target proteins with their respective top hit ligands: (**a**) CiaR–campesterol; (**b**) LepC–meliantrol; (**c**) OppC–stigmasterol; (**d**) SecA–citrostadienol; (**e**) SMU1784c–citrostadienol; (**f**) SpaR–isofucosterol; (**g**) Yidc2–ursolic acid.

**Figure 2:**
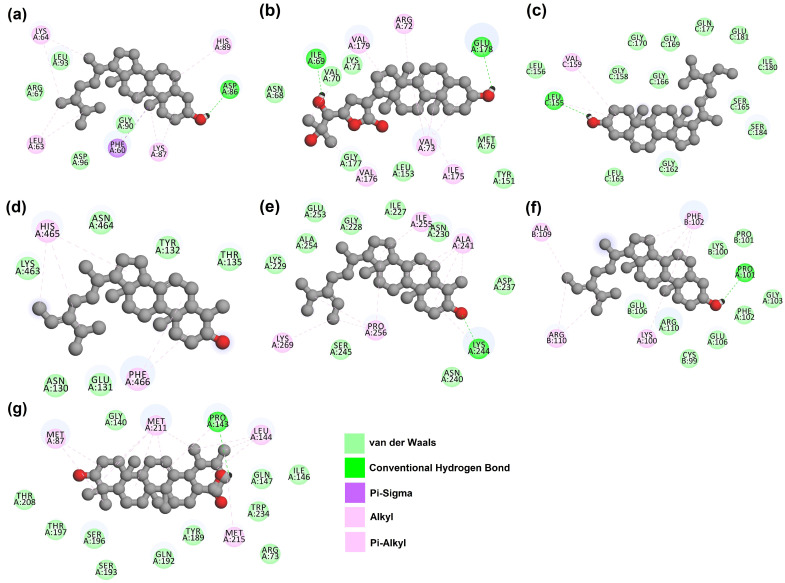
2D interaction of target proteins with their respective top hit ligands: (**a**) CiaR–campesterol; (**b**) LepC–meliantrol; (**c**) OppC–stigmasterol; (**d**) SecA–citrostadienol; (**e**) SMU1784c–citrostadienol; (**f**) SpaR–isofucosterol; (**g**) Yidc2–ursolic acid.

The 3D and 2D interactions between amino acid residues of the target proteins and their respective top hit compounds were also plotted using Discovery Studio. The list of amino acids present in each of the targets that interact with the ligands is presented in Table 3. The 3D and 2D plots depicting the interactions between target proteins and their ligands are presented in Figure 1 and Figure 2, respectively.

### 2.3. Molecular Dynamics (MD) Simulation

**Figure 3:**
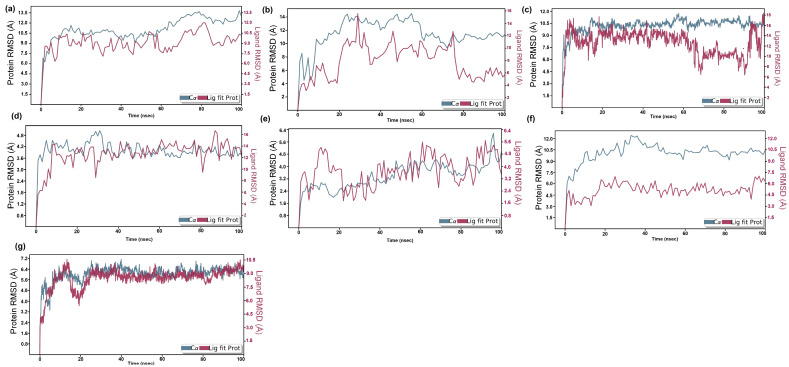
RMSD plot of protein–ligand complexes: (**a**) CiaR–campesterol; (**b**) LepC–meliantrol; (**c**) OppC–stigmasterol; (**d**) SecA–citrostadienol; (**e**) SMU1784c–citrostadienol; (**f**) SpaR–isofucosterol; (**g**) Yidc2–ursolic acid.

**Figure 4:**
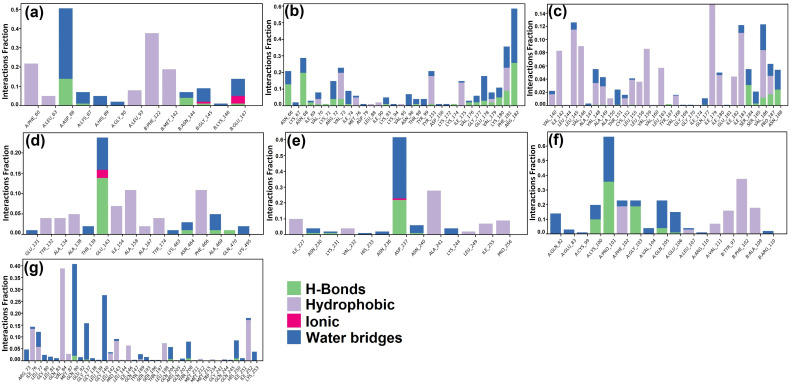
Hydrogen and hydrophobic interactions of protein–ligand complexes: (**a**) CiaR–campesterol; (**b**) LepC–meliantrol; (**c**) OppC–stigmasterol; (**d**) SecA–citrostadienol; (**e**) SMU1784c–citrostadienol; (**f**) SpaR–isofucosterol; (**g**) Yidc2–ursolic acid.

**Figure 5:**
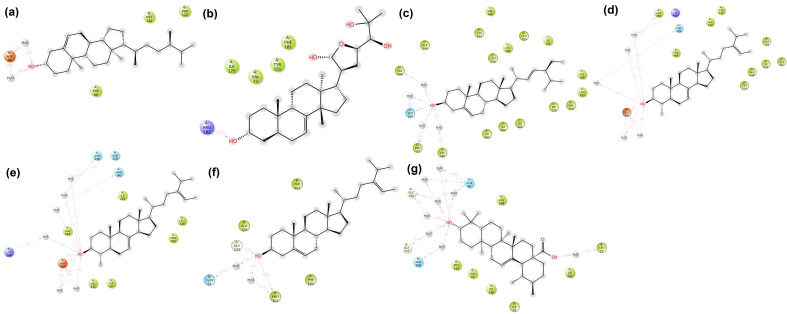
Protein–ligand contacts between target proteins and their respective ligands: (**a**) CiaR–campesterol; (**b**) LepC–meliantrol; (**c**) OppC–stigmasterol; (**d**) SecA–citrostadienol; (**e**) SMU1784c–citrostadienol; (**f**) SpaR–isofucosterol; (**g**) Yidc2–ursolic acid.

**Figure 6:**
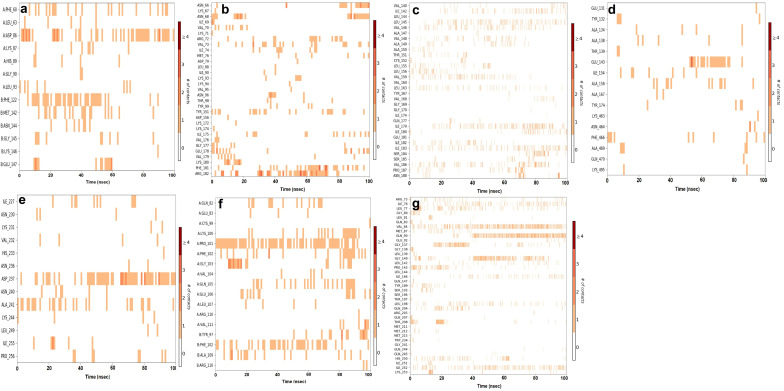
Timeline representation of interaction and contacts between target proteins and their respective ligands: (**a**) CiaR–campesterol; (**b**) LepC–meliantrol; (**c**) OppC–stigmasterol; (**d**) SecA–citrostadienol; (**e**) SMU1784c–citrostadienol; (**f**) SpaR–isofucosterol; (**g**) Yidc2–ursolic acid.

MD simulation assessed the inter-molecular protein–ligand interactions under an artificial environment with specified thermodynamical conditions such as temperature, volume, density, and pressure for 100 ns time duration. Further, a final production step aided in the exploration of the structural modification of the complex. The analysis of unique parameters such as root mean square deviation (RMSD) (Figure 3), simulation interactions diagram (Figure 4), protein–ligand contact (Figure 5), and timeline representation of interaction and contacts (Figure 6) between the target protein and their respective ligands, aided in the analysis of structural changes at each level.

The hydrogen bonding interaction between amino acid residues of the target protein and their respective ligand analyzed in MD simulation was found to correlate with that of AutoDock results (Table 3). The simulation study provided insight into the stability of the interaction between the top hits and their respective target proteins.

In order to perform the post-MM-GBSA (molecular mechanics with generalized Born and surface area solvation) analysis of the free binding energy calculation, 0-2002 frames with a sampling size of roughly 10 steps were produced. During the MM-GBSA calculation of the 100 ns MD data of the target proteins with their respective ligands, a total of 201 frames were processed and analyzed. All the complexes showed good binding affinity, thereby validating the docking and MD results (Table 4).

### 2.4. ADMET Analysis

**Figure 7:**
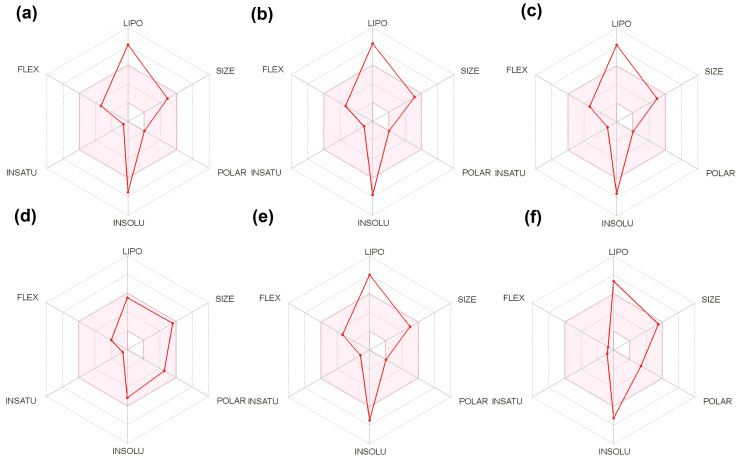
Bioavailability RADAR Plot analysis of the selected top hit ligands: (**a**) campesterol; (**b**) citrostadienol; (**c**) isofucosterol; (**d**) meliantrol; (**e**) stigmasterol; (**f**) ursolic acid.

**Figure 8:**
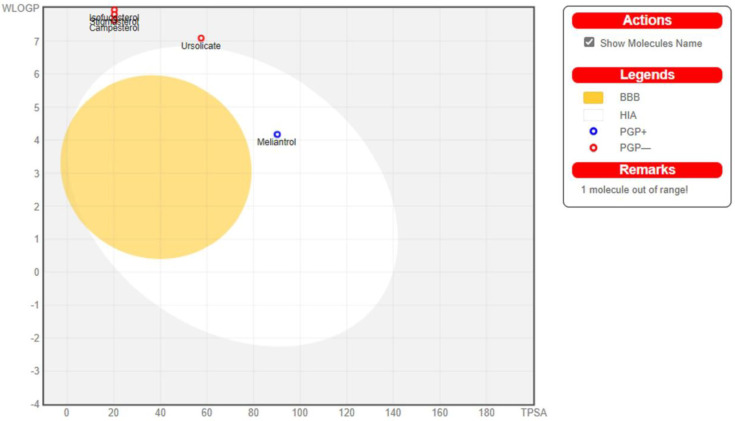
BOILED-Egg plot of selected high-binding ligands as predicted using SwissADME.

Analysis of ADMET properties of the top hits was performed using the SwissADME online tool and the pkCSM tool, and the results are presented in Table 5 and Table 6, respectively. All six compounds had molecular weights greater than 400 g/mol. Except for meliantrol, which was predicted to be moderately soluble in water, all other selected bioactive compounds were predicted to be poorly soluble in water. Solubility of ligands aid in the proper solvation and absorption into the host body. This also aids in the formulation of a solvent or carrier for delivery. Due to their poor solubility, all the hits violated one out of five of Lipinski’s rules. The RADAR plot (Figure 7) shows the overall drug-likeness of the top hit ligands. Meliantrol passes all the criteria for drug-likeness; however, other ligands violate the drug-likeness due to their poor solubility in water. The BOILED-Egg plot (Figure 8) shows all the compounds, except citrostadienol, in the area of intestinal absorption. However, only meliantrol was predicted to exhibit good GI absorption. None of the hits cross the blood–brain barrier. The results were correlated with that of pkCSM.

## 3. Discussion

*Streptococcus mutans* is one of the oral commensals that opportunistically cause dental caries. One of the pathogenic mechanisms adopted by *S. mutans* to cause dental caries is the formation of biofilm on the tooth surface which results in the erosion of tooth enamel through acid production. Biofilm formation requires quorum sensing, a density-dependent communication mechanism. The development of antimicrobial resistance in these pathogens can be avoided by using newer approaches rather than conventional antibiotics. These approaches should control the virulence of the pathogen instead of killing them. Hence, the objective of this study was to develop a compound that can control the quorum sensing in *S. mutans* rather than killing it. Pharmacoinformatics-based approaches such as drug target identification, molecular docking, and molecular dynamics simulations have accelerated the process of drug discovery. In the present study, we used bioinformatics tools and software to screen phytochemicals from *A. indica*, *M. citrifolia*, and *S. persica* for their potential as anti-quorum sensing agents against the selected target proteins of *S. mutans*.

The target proteins involved in quorum sensing were selected from our previous study [21]. These seven targets included CiaR (putative response regulator CiaR), LepC (signal peptidase I), OppC (putative transmembrane protein, permease OppC), SecA (protein translocase subunit SecA), SMU1784c (putative Eep protein-like protein), SMU_659 (putative response regulator SpaR), and YidC2 (membrane protein insertase YidC2). The target protein, CiaR, is a response regulator in a two-component signal transport system that controls several virulent characteristics of *S. mutans*. These virulent characteristics include mutacin I activity, oxidative stress tolerance, acid tolerance, and biofilm formation [22,23]. Studies on *S. sanguinis* showed the development of a fragile biofilm as a result of the CiaR mutation, which resulted in decreased polysaccharide synthesis [24]. The product of the lepC target gene is a signal peptidase that aids in the export of several virulent proteins. It has also been used as a housekeeping gene for several studies [10,25]. The target protein, OppC, is an oligopeptide permease of the ABC transporter family. It helps the bacteria to regulate XIP (sig X inducing peptide) production and in competence development [9,26]. SecA is a membrane protein translocase that helps the bacteria export proteins, leading to an increase in the virulence of the organism [10,27,28]. Another target, SMU1784c, plays an important role in the management of oxidative and acid stress, EPS production, and biofilm formation [11]. The SpaR protein is a response regulator of the spa (surface protein antigen) family, which is one of the virulence factors of *S. mutans* [28,29]. The membrane protein insertase of *S. mutans*, Yidc2, helps in EPS production and biofilm formation [10,30,31].

The resolved structures of the target proteins were not available in PDB or any other structural databases. Hence, the SWISS-MODEL online tool was used to predict the 3D structure of target molecules. Once the protein sequence is submitted as a query, SWISS-MODEL performs BLAST against PDB and gives a list of templates. Based on high identity and sequence coverage, five templates were selected for each protein, and the 3D models were built. A similar approach has been followed by researchers who have used SWISS-MODEL to model NOX 2 of *S. mutans* by using the crystal structure of NADH oxidase from *S. pyogenes* as a template [32,33]. Various proteins of *S. mutans* that were modeled using SWISS-MODEL include domain V of glucosyltransferase (GTF-SI) [34]; SMU.63, an amyloid-like secretory protein of *S. mutans* [35]; the Spase I protein and β-sheet-rich N-terminal collagen-binding domain (CBD) of Cnm, a collagen- and laminin-binding surface adhesin protein of *S. mutans* [36,37]. Since the models are predicted in silico, it requires validation before further processing. Hence, the Ramachandran plot from the structure assessment tool of SWISS-MODEL was used for validation of the predicted structures. All five models had more than 90% residues in the allowed region. The models from other templates with less than 90% residues in the allowed region were rejected. The remaining selected models were taken for further docking studies. The modeled structures of fibronectin/fibrinogen binding protein (FBP) from *S. mutans*, phospholipase D (F13) protein of monkeypox virus, 3-chymotrypsin and papain-like proteases of SARS-CoV2, and U box domain-containing protein gene (GsPUB8) from *Glycine soja* were all validated using the Ramachandran plot [38,39,40,41].

Computational drug discovery studies evaluate the binding of ligands with the target protein, but the agonist or antagonistic effect of ligands on the target protein is only validated through in vitro and in vivo experiments [42,43]. In the current study, based on an initial screening of 110 compounds using AutoDock, 17 high binders were selected that were found to bind with all the target proteins efficiently. Among the 17 compounds, 15 were from *A. indica*, and 1 each from *M. citrifolia* and *S. persica*. Campesterol, meliantrol, stigmasterol, isofucosterol, and ursolic acid were the top binders specific for the target proteins, CiaR, LepC, OppC, SpaR, and Yidc2, with a binding energy of −8.76, −10.16, −6.75, −9.1, and −9.73 kcal/mol, respectively. Citrostadienol was the high binder against two of the selected targets, SMU1784c and SecA, with a binding score of −8.45 and −9.88 kcal/mol, respectively. The source of ursolic acid is *M. citrifolia*, whereas all other leads are constituents of *A. indica*. Molecular docking is a versatile tool that is very useful in screening hundreds of compounds before testing the effective ones using in vitro studies. Molecular docking using AutoDock tools has been used previously to study both agonist and antagonist activity of various natural and synthetic ligands. In silico and in vitro agonistic activity of ligands have been studied for the treatment of diabetes [44,45], Parkinson’s disease [46], and cardiac diseases [47]. The antagonistic activity of ligands against *S. mutans* [48,49], *Leishmania donovani* [50], *Helicobacter pylori* [51], and SARS-CoV-2 [52] has also been studied. All these research works corroborate the necessity of in vitro experiments in the validation of computational analysis. However, they also demonstrate that phytocompounds and synthetic compounds can cause competitive or non-competitive inhibition of target proteins involved in microbial diseases. 

The RMSD of the protein–ligand complex is plotted to evaluate the stability of the interaction between the protein and the ligand. The RMSD plot of target protein CiaR in complex with its top binding ligand, campesterol, displayed a fluctuation in RMSD up to 7 Å to 13 Å (Figure 5a). Ligand RMSD was stable, and fluctuations were between 9 and 10.5 Å. The complex of LepC and its top hit ligand meliantrol shows RMSD fluctuation up to 8 and 12 Å (Figure 5b). Ligand RMSD was stable, and fluctuations were between 4 and 10.6 Å. The simulation of target protein OppC in complex with stigmasterol shows the stability of protein at 9 to 16 Å (Figure 5c). Ligand RMSD was stable, and fluctuations were between 9 and 1.6 Å. In the SecA–citrostadienol complex, the protein remains stable at 3 to 4 Å (Figure 5d), whereas the ligand fluctuation is at 14–16 Å and, hence, shows stable interaction between the protein and the ligand. The SMU1784c–citrostadienol complex shows fluctuation up to 2 to 4 Å (Figure 5e), whereas the ligand fluctuation is at 3–6 Å and, hence, shows stable interaction between the protein and the ligand. The Yidc2 and ursolic acid complex shows good RMSD results. The protein fluctuation is up to 6 Å and the ligand up to 9 Å. The RMSD plot converges from 20 ns till the end of the simulation at 100 ns, thus showing a stable interaction between the protein and the ligand. The hydrogen bond and other interactions plotted in the simulation interactions diagram correlate with the docking results. The hydrogen bond interaction between residues ASP86, LEU155, and PRO101 in CiaR, OppC, and SpaR with their respective ligands was observed both in AutoDock and MD simulations. Similarly, in the LepC protein, amino acid residues ILE69 and GLU178 were observed. In SecA, SMU1784c, and Yidc2 though there were no common residues binding through a hydrogen bond, the same residues bind with ligands through other types of bonds. The highly interacting amino acids were ASP86 of CiaR, ARG182 of LepC, ILE179 of OppC, GLU143 of SecA, ASP237 of SMU_1784c, PRO101 of SpaR, and VAL84 of YidC2. Similarly, molecular dynamic simulations and an energy calculation method have been used by researchers to study the LPXTG sequence in the C-terminus of surface proteins, the substrate of the cysteine transpeptidase sortase A (SrtA) enzyme, to better understand how leucine residue affects the dynamics of the enzyme-substrate complex structure. According to the findings, the substrate’s ‘Leu’ residue appears to be essential for anchoring and guiding the conformational shift of the enzyme SrtA [53]. Molecular docking and dynamics simulation studies have been exploited to study the inhibition of glucan sucrase-mediated biofilm formation of *S. mutans* by thiosemicarbazide derivatives [54]. Similar techniques have also been used to investigate the stability of phosphodiesterase type 5 (PDE5) in complexes with bioactive compounds from *Mimosa pudica* to understand their aphrodisiac performance [55]. Similarly, a pharmacoinformatics-based molecular docking and dynamics simulation analysis of bioactive components from Indian cuisine, rasam, was conducted against MAPK6 (mitogen-activated protein kinase 6), a family of serine/threonine protein kinases that is crucial in regulating extracellular signaling into a variety of cellular functions, including ROS production [56].

MM-GBSA also validates the molecular docking and MD simulation studies as it shows binding energy ranging from −49 to −79 kcal/mol in all the protein–ligand complexes studied. MM-GBSA has previously been used to study and validate in silico interaction of ligands with SARS-CoV-2 protease [57]. A similar MD simulation approach has also been utilized for screening substrate analog inhibitors of L-Ornithine-N5-monooxygenase (PvdA) to control *Pseudomonas aeruginosa* infections [58].

Analysis of ligands for ADMET properties using SwissADME and pkCSM shows that the molecular weight of all six hits was larger than 400g/mol. Only meliantrol was expected to have moderate water solubility. All other hits had low water solubility. The right solvation and absorption into the host body are made possible by the ligands’ solubility. Additionally, it helps with the formulation of the solvent or delivery vehicle. Except for meliantrol, none of the selected compounds exhibited good GI absorption. All the hits broke one out of five of Lipinski’s rules because of their poor solubility. The Bioavailability RADAR plot depicts the overall drug-likeness of the top binding ligands. Meliantrol fulfills all criteria for drug-likeness, while other ligands fail due to their low solubility in water. The BOILED-Egg plot displays all of the hits in the area of intestinal absorption except citrostadienol. None of the ligands were predicted to penetrate the blood–brain barrier. Analysis of ADMET properties of an array of ligands using Swiss ADME and pkCSM tools has been previously followed by many researchers to control *S. mutans* biofilm [59,60].

## 4. Materials and Methods

### 4.1. Target Proteins in S. mutans

The target proteins were selected based on our earlier study, which demonstrated an in silico subtractive proteomics approach for screening drug targets in *S. mutans* UA159. Among the 13 novel drug targets that were identified, 7 proteins were found to be involved in quorum sensing [21]. Hence, these target proteins have been subsequently used in the current study. The target proteins include two putative response regulators, a signal peptidase, a putative EEP protein-like protein, a putative transmembrane protein-permease, a protein translocase subunit, and a membrane protein insertase (Table 7). 

### 4.2. Selection of Ligands

The bioactive compounds from three Indian medicinal plants, *Azadirachta indica*, *Morinda citrifolia*, and *Salvodora persica,* were selected as ligands from the literature, as well as various databases such as Dr. Duke’s Phytochemical and Ethnobotanical Databases (https://phytochem.nal.usda.gov/; accessed on 11 April 2022), IMPPAT (https://cb.imsc.res.in/imppat/; accessed on 13 April 2022), PubChem (https://pubchem.ncbi.nlm.nih.gov/; accessed on 15 April 2022), and Drug Bank (https://go.drugbank.com/; accessed on 18 April 2022). However, among the hundreds of compounds, the ones with a background of antimicrobial activity in the literature were selected. The screening process involved manually searching each compound in the PubMed database for its antimicrobial activity against *S. mutans* or other oral pathogens. Finally, 110 compounds were shortlisted for analysis. The 3D structures of these ligands were either collected from PubChem or drawn using ChemSketch Version 12.00 (Table 8).

### 4.3. Computational Analysis 

Computational analysis was performed using Debian Linux Operating System running on a 3.10 GHz Intel^®^ Core™ i5-4440 CPU (Acer, Bengaluru, India) with 8 GB RAM. For the analysis of docking results, the Accelrys^®^ Discovery Studio Visualizer (Accelrys Software Inc., San Diego, CA, USA) that runs on the Windows operating system was used.

### 4.4. Homology Modeling and Model Validation

The 3D structures of target proteins were modeled using the SWISS-MODEL online tool (https://swissmodel.expasy.org/ accessed on 20 April 2022) [61]. The modeled structures were assessed and validated using the Ramachandran plot analysis.

### 4.5. Molecular Docking

The target proteins were prepared by removing the water molecules using Discovery Studio. The parameters for docking were set up by using the graphical user interface (GUI) of AutoDock Tools [62]. The preparation of the target protein structure involves the addition of hydrogen atoms and formal charges. The number of rotatable bonds and determination of root for the ligand were set to default. Later, molecular docking was performed using AutoDock 4.2. The AutoGrid4 program of AutoDock allowed the generation of grid maps for target proteins embedded in a three-dimensional grid of manually set parameters (Table 9).

The binding energies between the target protein and ligand were calculated by running the AutoDock4 program using pre-set grid maps. The result analysis was performed using the GUI of AutoDock Tools and Discovery Studio. The binding energies for various conformations of the ligand with the target proteins were determined, and the best conformation was chosen based on the binding energy and the number of hydrogen bonds that they formed with the protein.

### 4.6. Molecular Dynamics

The protein–ligand complexes (PLCs) were prepared using PyMOL [63], and these PLCs were taken as input for Molecular Dynamics (MD) simulations (Table 10). The MD simulation of PLCs aids in the visualization of target binding sites and also provides information concerning the binding stability of the PLCs under physiological conditions. The Desmond module of Schrödinger was used to perform MD simulations (academic license, Version 2020-1) [64]. Initially, an explicit water model was prepared as an orthorhombic simulation box with Simple Point-Charge (SPC). This was designed with the builder panel of the system in such a way that a minimum distance of 10 Å is maintained between the protein surface and the solvent surface. Then, the PLCs with receptors were solvated with the orthorhombic TIP3P water model [65]. The addition of counter ions and limitation of salt concentration of the physiological system to 0.15M was performed for the neutralization of the solvated system (Table 11).

Then, the PLC system was designated with the OPLS AA force field [66]. For the RESPA (reversible reference system propagator algorithms) integrator [67], Nose–Hoover chain thermostat [68], and Martyna–Tobias–Klein barostat, a relaxation time of two seconds was used. The equilibrated system was used to perform the final production process of MD simulations. In this study, the default parameters set for relaxation before MD simulations include a time duration of 100 ns, temperature of 310 K, a pressure of 1.0 bar with NPT (Isothermal–Isobaric ensemble, constant temperature, constant pressure, constant number of particles) ensemble [69] (Table 12).

The MD simulation tool was used to run the simulation, and the output file was used to retrieve trajectories and create the movie. The output file in .cms format was imported into the software, and the created simulation movie was exported at a higher resolution of 1280 × 1024 px for better quality. The trajectory of the overall MD simulation process was written in 1000 frames. The frames of the protein backbone were aligned to the backbone of the first frame. This provides a better understanding of the stability of the PLCs during MD simulations. Finally, the Root Mean Square Deviation (RMSD) and Root Mean Square Fluctuation (RMSF) of PLCs and the simulation interaction diagrams were analyzed [57,70]. For each protein–ligand complex, three simulations studies were run, and the best run was taken for analysis.

The free binding energies of the protein and ligand complexes were also examined using Molecular Mechanics, the Generalized Born model, and Solvent Accessibility (MM-GBSA). The “thermal_MMGBSA.py” script and “MM-GBSA ΔG Bind:” parameter from the Prime/Desmond module of the Schrodinger suite were employed to carry out the post-MM-GBSA analysis [71].

### 4.7. ADMET Analysis

The ADMET properties of the high binding ligands were analyzed using the SwissADME online tool (http://www.swissadme.ch; accessed on 10 May 2022) [72] and pkCSM online tools (http://biosig.unimelb.edu.au/pkcsm/prediction; accessed on 10 May 2022) [73]. The input was given as SMILES notation of the ligands, and the output was retrieved as a “.csv” file. In SwissADME, the BOILED-Egg plot and radar plot for all the leads were also retrieved as a single image [74]. The ADMET analysis helps to predict the drug-likeness of the top hits.

## 5. Conclusions

*S. mutans* is a major contributor to dental caries. In this study, an in silico approach was employed to identify a plant metabolite with potential efficacy against specific target proteins of *S. mutans* involved in quorum sensing. The selected target proteins included: Membrane protein insertase YidC 2, Permease OppC, Putative Eep protein-like protein, Putative transmembrane protein, Putative response regulator CiaR, Putative response regulator SpaR, and Signal peptidase I LepC. The three-dimensional models of these target proteins were generated using SWISS-MODEL and validated using the Ramachandran plot. Plant metabolites derived from *A. indica* (Neem), *M. citrifolia* (Noni), and *S. persica* (Miswak) were evaluated for their potential binding affinity to the selected target proteins. Molecular docking studies were performed using AutoDock Tools. From a total of 110 ligands, 6 top hits were identified for each target protein. These ligands, namely campesterol, meliantrol, citrostadienol, stigmasterol, isofucosterol, and ursolic acid, were subjected to molecular simulation analysis to assess their stability and interaction patterns. The highly interacted amino acid residues identified were ASP86, ARG182, ILE179, GLU143, ASP237, PRO101, and VAL84, corresponding to the proteins CiaR, LepC, OppC, SecA, SMU1784c, SpaR, YidC2, respectively. Furthermore, the ADME characteristics of the identified ligands were evaluated using the SwissADME program. Collectively, the results suggest that these phytosterols have the potential to serve as quorum-quenching agents. However, further in vitro analysis is required to confirm their efficacy against *S. mutans* and to enable their application in the treatment of dental caries.

## Figures and Tables

**Table 1:** List of top hits from different plant sources against each target protein, along with their binding energy (kcal/mol).

Ligands	CIAR	LEPC	SMU1784C	OPPC	SPAR	SECA	YIDC2	Plant Source
5α-Androstan-16-one, cyclic ethylene mercaptole	−7.47	−9.5	−7.46	−6.23	−8.29	−9.26	−8.24	*S. persica*
Azadiradione	−7.91	−9.18	−8.32	−6.22	−8.1	−8.45	−8.23	*A. indica*
Azadirone	−7.81	−8.85	−8.34	−5.83	−8.34	−8.16	−8.85	*A. indica*
Campesterol	−8.76	−9.91	−8.08	−6.41	−8.56	−9.29	−8.86	*A. indica*
Citrostadienol	−7.55	−9.81	−8.45	−6.43	−8.9	−9.88	−9.4	*A. indica*
Isofucosterol	−8.44	−9.31	−8.14	−6.68	−9.1	−9.58	−8.94	*A. indica*
Margocin	−7.24	−8.85	−7.58	−5.94	−8.11	−7.81	−8.07	*A. indica*
Meliantrol	−8.44	−10.16	−7.43	−5.95	−8.14	−8.66	−8.61	*A. indica*
Nimbinene	−7.41	−7.45	−7.88	−5.38	−7.39	−7.99	−7.39	*A. indica*
Nimbione	−7.09	−7.61	−7.63	−5.91	−7.65	−7.7	−7.46	*A. indica*
Nimbolide	−7.62	−8.14	−7.57	−5.8	−8.45	−7.56	−8.3	*A. indica*
Nimbolin b	−7.9	−9.09	−7.58	−6.09	−9.07	−9.77	−7.41	*A. indica*
Nimocinol	−7.95	−8.55	−8.27	−5.98	−8.59	−7.84	−8.41	*A. indica*
Stigmasterol	−7.78	−9.87	−8.35	−6.75	−8.76	−9.12	−9.04	*A. indica*
Ursolic acid	−7.98	−9.42	−8.31	−6.12	−8.14	−7.54	−9.73	*M. citrifolia*
Vepinin	−7.81	−10.06	−7.69	−5.51	−7.85	−9	−8.38	*A. indica*
Zafaral	−7.71	−9.12	−7.3	−5.88	−7.8	−8.09	−8.1	*A. indica*

**Table 2:** List of top hits from different plant sources against each target protein, along with their binding energy.

Top Binders	Binding Energy (kcal/mol)	Target
Campesterol	−8.76	CIAR
Meliantrol	−10.16	LEPC
Citrostadienol	−8.45	SMU1784C
Stigmasterol	−6.75	OPPC
Isofucosterol	−9.1	SPAR
Citrostadienol	−9.88	SECA
Ursolic acid	−9.73	YIDC2

**Table 3:** List of amino acid residues interacting with the ligand. The residues which interact with ligands through conventional hydrogen bonds during the docking process are bold-faced.

Amino Acid Residues Interacting through Hydrogen Bond	
CiaR	**ASP86**; LYS87; ASN144; GLY145; GLU147
LepC	ASN66; ASN68; **ILE69**; VAL70; LYS71; ARG72; VAL73; ILE74; LYS93; THR98; TYR151; LYS174; VAL176; GLY177; **GLU178**; VAL179; LYS180; PHE181; ARG182
OppC	VAL148; ALA149; THR151; **LEU155**; TYR17; GLN177; SER184; SER185; VAL186; PRO187; ASN188
SecA	GLU143; ASN464; ALA469; GLN470
SMU1784c	ASN230; LYS231; ASP237; ASN240
SpaR	LYS100; **PRO101**; GLY103; GLN105; GLU106
Yidc2	ARG73; LEU77; LEU81; GLN90; GLY137; GLN204; THR208; HIS250

**Table 4:** MMGBSA Score of Protein–Ligand Complexes.

Protein–Ligand Complex	MMGBSA Score (kcal/mol)
CiaR–campesterol	−59.03099478
LepC–meliantrol	−49.87694241
OppC–stigmasterol	−65.96184631
SecA–citrostadienol	−60.69835805
SMU1784c–citrostadienol	−57.96969001
SpaR–isofucosterol	−58.58904881
Yidc2–ursolic acid	−79.57923832

**Table 5:** ADME properties of top hits assessed in SWISS-ADME.

Molecule	Campesterol	Citrostadienol	Isofucosterol	Meliantrol	Stigmasterol	Ursolic Acid
Physicochemical Properties						
Formula	C_28_H_48_O	C_30_H_50_O	C_29_H_48_O	C_28_H_46_O_5_	C_29_H_48_O	C_30_H_48_O_3_
Molecular Weight	400.68	426.72	412.69	462.66	412.69	456.7
#Heavy atoms	29	31	30	33	30	33
#Aromatic heavy atoms	0	0	0	0	0	0
Fraction Csp3	0.93	0.87	0.86	0.93	0.86	0.9
#Rotatable bonds	5	5	5	3	5	1
#H-bond acceptors	1	1	1	5	1	3
#H-bond donors	1	1	1	4	1	2
MR	128.42	137.56	132.75	130.66	132.75	136.91
TPSA	20.23	20.23	20.23	90.15	20.23	57.53
Lipophilicity						
iLOGP	4.92	5.41	5.15	3.76	5.01	3.71
XLOGP3	8.8	9.03	8.85	4.18	8.56	7.34
WLOGP	7.63	8.19	7.94	4.17	7.8	7.09
MLOGP	6.54	6.82	6.62	3.45	6.62	5.82
Silicos-IT Log P	6.63	7	6.88	3.46	6.86	5.46
Consensus Log P	6.9	7.29	7.09	3.8	6.97	5.88
Water Solubility						
ESOL Log S	−7.54	−7.84	−7.64	−5.14	−7.46	−7.23
ESOL Solubility (mg/mL)	0.0000116	0.0000061	0.00000936	0.00332	0.0000143	0.0000269
ESOL Solubility (mol/l)	0.000000029	0.0000000143	0.0000000227	0.00000718	0.0000000346	0.0000000589
ESOL Class	Poorly soluble	Poorly soluble	Poorly soluble	Moderately soluble	Poorly soluble	Poorly soluble
Ali Log S	−9.11	−9.35	−9.16	−5.78	−8.86	−8.38
Ali Solubility (mg/mL)	0.000000313	0.000000192	0.000000286	0.000764	0.000000571	0.00000192
Ali Solubility (mol/L)	0.00000000078	0.00000000045	0.000000000692	0.00000165	0.00000000138	0.00000000421
Ali Class	Poorly soluble	Poorly soluble	Poorly soluble	Moderately soluble	Poorly soluble	Poorly soluble
Silicos-IT LogSw	−5.79	−5.97	−5.83	−3.14	−5.47	−5.67
Silicos-IT Solubility (mg/mL)	0.000642	0.000457	0.000616	0.332	0.0014	0.000972
Silicos-IT Solubility (mol/L)	0.0000016	0.00000107	0.00000149	0.000718	0.00000339	0.00000213
Silicos-IT class	Moderately soluble	Moderately soluble	Moderately soluble	Soluble	Moderately soluble	Moderately soluble
Pharmacokinetics						
GI absorption	Low	Low	Low	High	Low	Low
BBB permeant	No	No	No	No	No	No
Pgp substrate	No	No	No	Yes	No	No
CYP1A2 inhibitor	No	No	No	No	No	No
CYP2C19 inhibitor	No	No	No	No	No	No
CYP2C9 inhibitor	No	No	No	No	Yes	No
CYP2D6 inhibitor	No	No	No	No	No	No
CYP3A4 inhibitor	No	No	No	No	No	No
log Kp (cm/s)	−2.5	−2.49	−2.53	−6.15	−2.74	−3.87
Druglikeness						
Lipinski #violations	1; MLOGP > 4.15	1; MLOGP > 4.15	1; MLOGP > 4.15	0	1; MLOGP > 4.15	1; MLOGP > 4.15
Ghose #violations	2; WLOGP > 5.6, #atoms > 70	3; WLOGP > 5.6, MR > 130, #atoms > 70	3; WLOGP > 5.6, MR > 130, #atoms > 70	2; MR > 130, #atoms > 70	3; WLOGP > 5.6, MR > 130, #atoms > 70	3; WLOGP > 5.6, MR > 130, #atoms > 70
Veber #violations	0	0	0	0	0	0
Egan #violations	1; WLOGP > 5.88	1; WLOGP > 5.88	1; WLOGP > 5.88	0	1; WLOGP > 5.88	1; WLOGP > 5.88
Muegge #violations	2; XLOGP3 > 5, Heteroatoms < 2	2; XLOGP3 > 5, Heteroatoms < 2	2; XLOGP3 > 5, Heteroatoms < 2	0	2; XLOGP3 > 5, Heteroatoms < 2	1; XLOGP3 > 5
Bioavailability Score	0.55	0.55	0.55	0.55	0.55	0.85
Medicinal Chemistry						
PAINS #alerts	0	0	0	0	0	0
Brenk #alerts	1; isolated_alkene	1; isolated_alkene	1; isolated_alkene	1; isolated_alkene	1; isolated_alkene	1; isolated_alkene
Leadlikeness #violations	2; MW > 350, XLOGP3 > 3.5	2; MW > 350, XLOGP3 > 3.5	2; MW > 350, XLOGP3 > 3.5	2; MW > 350, XLOGP3 > 3.5	2; MW > 350, XLOGP3 > 3.5	2; MW > 350, XLOGP3 > 3.5
Synthetic Accessibility	6.17	6.22	6.15	6.71	6.21	6.21

# denotes number.

**Table 6:** ADMET properties of top hit ligands assessed in pkCSM.

Descriptor		Predicted Value						Unit
		**Campesterol**	**Citrostadienol**	**Isofucosterol**	**Meliantrol**	**Stigmasterol**	**Ursolic acid**	
	Molecular Weight	400.691	426.729	412.702	462.671	412.702	456.711	g/mol
	LogP	7.6347	8.1909	7.9449	4.1717	7.8008	7.0895	
	#Rotatable Bonds	5	5	5	3	5	1	
	#Acceptors	1	1	1	5	1	2	
	#Donors	1	1	1	4	1	2	
	Surface Area	180.674	192.714	186.349	199.164	186.349	201.354	
Property	Model Name							
Absorption	Water solubility	−7.194	−6.74	−6.917	−4.096	−6.882	−3.193	Numeric (log mol/L)
Absorption	CaCO2 permeability	1.284	1.285	1.279	1.526	1.28	1.286	Numeric (log Papp in 10^−6^ cm/s)
Absorption	Intestinal absorption (human)	95.749	95.907	96.061	97.238	96.39	100	Numeric (% Absorbed)
Absorption	Skin Permeability	−2.756	−2.691	−2.7	−2.726	−2.702	−2.732	Numeric (log Kp)
Absorption	P-glycoprotein substrate	No	Yes	No	Yes	No	No	Categorical (Yes/No)
Absorption	P-glycoprotein I inhibitor	Yes	Yes	Yes	Yes	Yes	No	Categorical (Yes/No)
Absorption	P-glycoprotein II inhibitor	Yes	Yes	Yes	Yes	Yes	No	Categorical (Yes/No)
Distribution	VDss (human)	0.351	−0.028	0.104	−0.074	0.102	−1.104	Numeric (log L/kg)
Distribution	Fraction unbound (human)	0	0	0	0	0	0	Numeric (Fu)
Distribution	BBB permeability	0.804	0.816	0.807	−0.848	0.814	−0.182	Numeric (log BB)
Distribution	CNS permeability	−1.43	−1.232	−1.326	−1.84	−1.326	−1.118	Numeric (log PS)
Metabolism	CYP2D6 substrate	No	No	No	No	No	No	Categorical (Yes/No)
Metabolism	CYP3A4 substrate	Yes	Yes	Yes	Yes	Yes	Yes	Categorical (Yes/No)
Metabolism	CYP1A2 inhibitor	No	No	No	No	No	No	Categorical (Yes/No)
Metabolism	CYP2C19 inhibitor	No	No	No	No	No	No	Categorical (Yes/No)
Metabolism	CYP2C9 inhibitor	No	No	No	No	No	No	Categorical (Yes/No)
Metabolism	CYP2D6 inhibitor	No	No	No	No	No	No	Categorical (Yes/No)
Metabolism	CYP3A4 inhibitor	No	No	No	No	No	No	Categorical (Yes/No)
Excretion	Total Clearance	0.572	0.585	0.619	0.446	0.618	0.083	Numeric (log mL/min/kg)
Excretion	Renal OCT2 substrate	No	No	No	No	No	No	Categorical (Yes/No)
Toxicity	AMES toxicity	No	No	No	No	No	No	Categorical (Yes/No)
Toxicity	Max. tolerated dose (human)	−0.193	−0.394	−0.374	−0.726	−0.385	0.239	Numeric (log mg/kg/day)
Toxicity	hERG I inhibitor	No	No	No	No	No	No	Categorical (Yes/No)
Toxicity	hERG II inhibitor	Yes	Yes	Yes	No	Yes	No	Categorical (Yes/No)
Toxicity	Oral Rat Acute Toxicity (LD50)	2.355	2.96	2.847	4.488	2.836	2.81	Numeric (mol/kg)
Toxicity	Oral Rat Chronic Toxicity (LOAEL)	1.125	1.096	1.119	2.249	1.102	2.128	Numeric (log mg/kg_bw/day)
Toxicity	Hepatotoxicity	No	No	No	No	No	Yes	Categorical (Yes/No)
Toxicity	Skin Sensitisation	No	No	No	No	No	No	Categorical (Yes/No)
Toxicity	*T. pyriformis* toxicity	0.676	0.405	0.48	0.288	0.481	0.285	Numeric (log ug/L)
Toxicity	Minnow toxicity	−2.071	−2.047	−1.988	2.749	−1.952	−1.204	Numeric (log mM)

# denotes number.

**Table 7:** List of selected target proteins involved in quorum sensing.

UniProt Id.	Gene Name	Protein Name
Q8DU28	CiaR	Putative response regulator CiaR
Q8DSC7	LepC	Signal peptidase I
Q8DW23	OppC	Putative transmembrane protein, permease OppC
Q8DSF0	SecA	Protein translocase subunit SecA
Q8DSJ8	SMU1784c	Putative Eep protein-like protein
Q8DV49	SMU659	Putative response regulator SpaR
Q8DSP8	YidC2	Membrane protein insertase YidC 2

**Table 8:** List of ligands selected from three plants, *A. indica*, *M. citrifolia*, and *S. persica*.

*Azadirachta indica*		
6-desacetyllnimbinene	Margocin	Oleic acid
Arachidic acid	Meliacinanhydride	Palmitic acid
Azadirachtin A	Meliantrol	Palmitoleic acid
Azadirachtin B	Nimbandiol	Quercetin
Azadiradione	Nimbidiol	Salanin
Azadirone	Nimbin	Stearic acid
Behenic acid	Nimbinene	Stigmasterol
Beta-sitosterol	Nimbinone	Vepinin
Campesterol	Nimbione	Vilasinin
Citrostadienol	Nimbolicin	Zafaral
Fraxinellone	Nimbolide	α-tocopherol
Gadoleic acid	Nimbolin A	β-tocopherol
Isomeldenin	Nimbolin B	γ-tocopherol
Linoleic acid	Nimocinol	Isofucosterol
Linolenic acid	Nimocinolide	δ-tocopherol
*Morinda citrifolia*		
2-methoxy-1,3,6-trihydroxyanthraquinone	Deacetylasperuloside	Nordamnacanthal
6R-hydroxyadoxoside	Dehydromethoxygaertneroside	Octanoic acid (caprylic acid)
Americanin A	Epi-dihydrocornin	Retinol
Ascorbic acid	L-asperuloside	Rubiadin
Asperulosidic acid	Methyl alpha-d-fructofuranoside	Rubiadin-1-methyl ether
Borreriagenin	Methyl beta-d-fructofuranoside	Rutin
Caproic acid	Morindacin	Scopoletin
Citrifolinin B epimer a	Morindone	Ursolic acid
Citrifolinin B epimer b	Narcissoside	
Citrifolinoside	Nicotifloroside	
*Salvadora persica*		
1H-Pyrazole-1-carbothioamide	Catechin	Pyrrolidine
1-triacantanol	Cineole	Salvadoside
3, 5-Dithiahexanol 5, 5-dioxide	Epicatechin	Salvadourea
5-O-caffeoylquinic acid	Farnesol	Syringin
5α-Androstan-16-one, cyclic ethylene mercaptole	Glycerin	Theobromine
Benzyl urea	Humulene	Trigonelline
Beta-sitosteryl arabinosyl vanilloyl stearate	Kaempferol	α-thujones
Borneol	Limonene	β-cymene
Bornyl acetate	Linalool	β-myrcene
Butanediamide	Naringenine	β-santatol
Caffeine	N-benzyl- 2-phenylacetamide	β-thujones
Camphor	N-benzyl-benzamide	
Caryophyllene	Octacosanol	

**Table 9:** List of parameters used to generate grid on target proteins for molecular docking.

Gene Name	No. of Grid Points in Axes (X, Y, Z)	No. of Grid Points in Å	Grid Center Coordinates (X, Y, Z)
CiaR	260, 160, 300	0.375	49.202, 21.493, −15.155
LepC	150, 250, 200	0.375	32.025, 56.24, 14.572
OppC	150, 100, 120	0.375	67.03, 139.688, 246.646
SecA	300, 300, 300	0.375	−7.808, 13.046, 09.175
SMU1784c	100, 100, 120	0.375	−30.648, 25.537, 8.584
SMU659 (SpaR)	250, 260, 300	0.375	−56.159, 24.702, −77.258

**Table 10:** Details of Protein–Ligand Complex (PLC).

Protein Name	CiaR	LepC	OppC	SecA	SMU1784c	SpaR	Yidc2
Total Residues	446	130	49	758	83	434	219
Protein Chain(s)	A, B	A	C	A	A	A, B	A
Residue in Chain(s)	223, 223	130	49	758	83	217, 217	219
No. of Atoms	7188	2104	746	12072	1324	7122	3509
No. of Heavy Atoms	3572	1043	356	6059	633	3520	1710
Atoms Charge	−24	9	−1	−24	14	4	9
Ligand name	Campesterol	Meliantrol	Stigmasterol	Citrostadienol	Citrostadienol	Isofucosterol	Ursolic acid
No. of Atoms	77 (total) 29 (heavy)	79 (total) 33 (heavy)	78 (total) 30 (heavy)	81 (total) 31 (heavy)	81 (total) 31 (heavy)	78 (total) 30 (heavy)	81 (total) 33 (heavy)
Atomic Mass [a.u.]	400.694	462.676	412.705	426.732	426.732	412.705	456.715
Molecular Formula	C_28_H_48_O	C_28_H_46_O_5_	C_29_H_48_O	C_30_H_50_O	C_30_H_50_O	C_29_H_48_O	C_30_H_48_O_3_
No. of Fragments	1	1	2	2	2	2	1
No. of Rotatable Bonds	6	7	6	6	6	6	3

**Table 11:** Details of counterions and salt concentration added for simulation.

Protein–Ligand Complex	Type	No. of Atoms	Concentration [mM]	Total Charge
CiaR–campesterol	Na	92	68.698	92
	Cl	68	50.777	−68
LepC–meliantrol	Na	32	50.492	32
	Cl	41	64.693	−41
OppC–stigmasterol	Na	1	4.283	1
	Cl	-	-	-
SecA–citrostadienol	Na	101	66.292	101
	Cl	77	50.54	−77
SMU1784c–citrostadienol	Na	12	50.811	12
	Cl	26	110.09	−26
SpaR–isofucosterol	Na	55	50.916	55
	Cl	59	54.62	−59
Yidc2–ursolic acid	Na	-	-	-
	Cl	9	15.519	−9

**Table 12:** Details of default parameters set before simulation.

Protein–Ligand Complex	Ensemble	Temperature [K]	Simulation Time [ns]	No. of Atoms	No. of Water Molecules	Charge
CiaR_campesterol	NPT	310.1	100.102	80,472	24,349	0
LepC_meliantrol	NPT	310.1	101	36,825	11,523	0
OppC_stigmasterol	NPT	310.1	100.102	13,560	4245	0
SecA_citrostadienol	NPT	310.1	101	95,434	27,701	0
SMU1784c_citrostadienol	NPT	310.1	101	14,325	4294	0
SpaR_isofucosterol	NPT	310.1	101	66,234	19,640	0
Yidc2_ursolic acid	NPT	310.1	100.102	35,231	10,544	0

## Data Availability

Additional data may be available upon personal request.

## Supplementary Materials

The following supporting information can be downloaded at: https://www.mdpi.com/article/10.3390/molecules28145514/s1. Figure S1: Ramachandran plot of modelled target proteins using structure assessment tool of the SWISS-MODEL: (a) ciaR; (b) lepC; (c) oppC; (d) secA; (e) smu1784c; (f) spaR and (g) yidc2, (h) represents the transmembrane localization of target protein yidc2. Figure S2: 3D structure of target proteins predicted using SWISS-MODEL online tool: (a) ciaR; (b) lepC; (c) oppC; (d) secA; (e) smu1784c; (f) spaR and (g) yidc2.
