# Supplementary material for: Pharmacoinformatics-Based Approach for Uncovering the Quorum-Quenching Activity of Phytocompounds against the Oral Pathogen, Streptococcus mutans
Source: Molecules. 2023 Jul 19;28(14):5514. doi: 10.3390/molecules28145514 (PMC10383507; doi:10.3390/molecules28145514)
Supplement: Supplementary file 1 [file molecules-28-05514-s001.zip › molecules-2471070-supplementary.pdf]

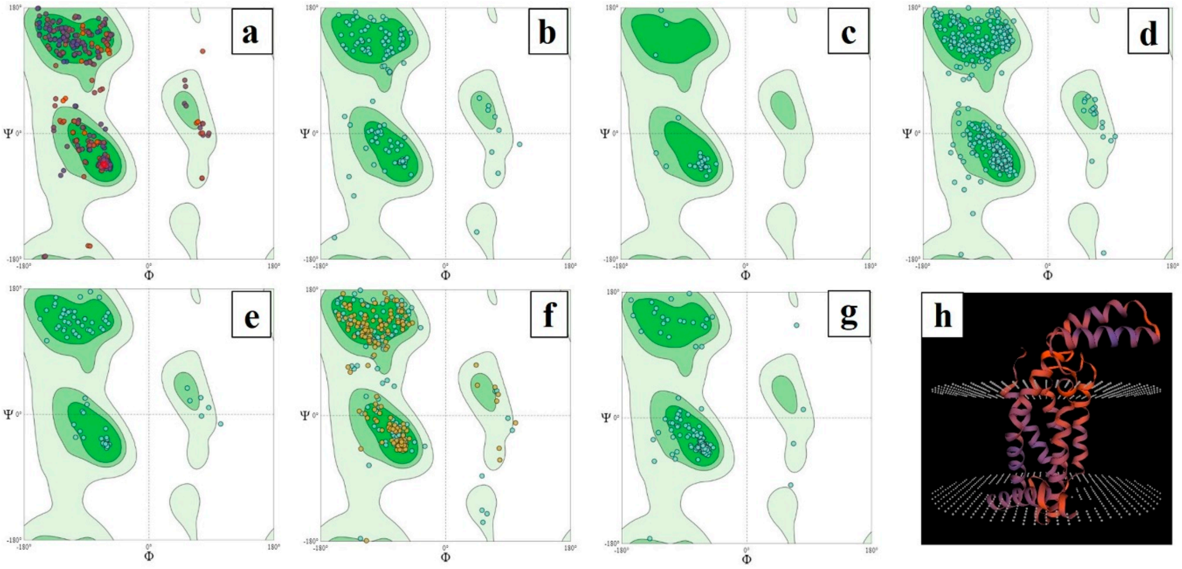

Figure S1. Ramachandran plot of modelled target proteins using structure assessment tool of the SWISS-MODEL: (**a**) ciaR; (**b**) lepC; (**c**) oppC; (**d**) secA; (**e**) smu1784c; (**f**) spaR and (**g**) yidc2. Figure (**h**) represents the transmembrane localization of target protein yidc2.

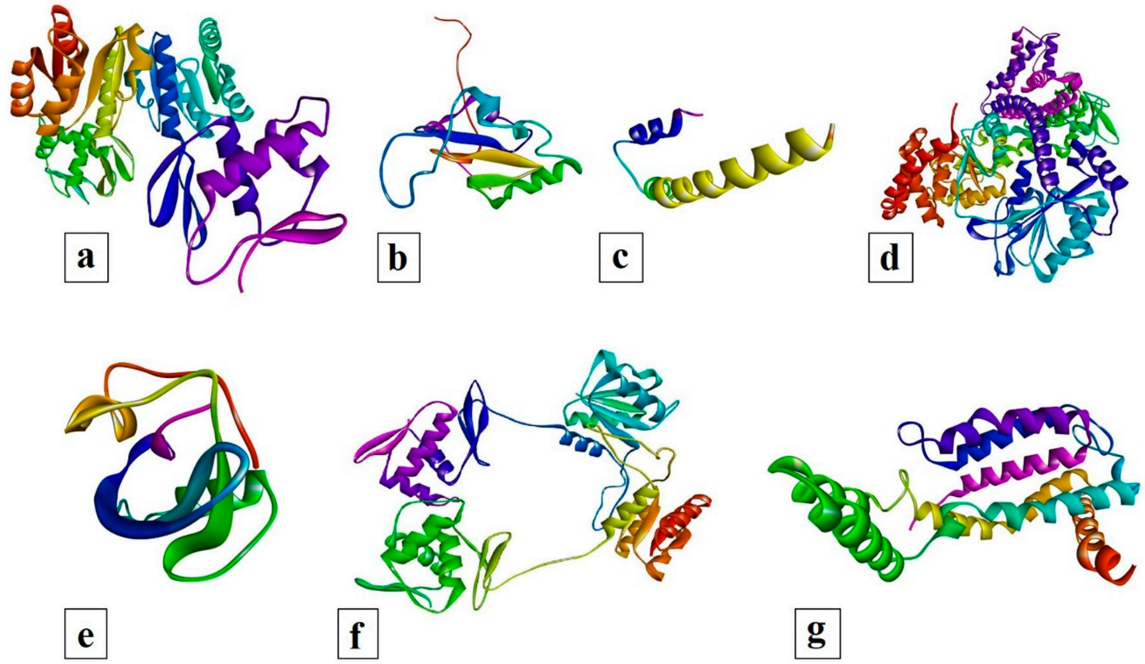

Figure S2 3D structure of target proteins predicted using SWISS-MODEL online tool: (**a**) ciaR; (**b**) lepC; (**c**) oppC; (**d**) secA; (**e**) smu1784c; (**f**) spaR and (**g**) yidc2.
